# *GmPAP12* Is Required for Nodule Development and Nitrogen Fixation Under Phosphorus Starvation in Soybean

**DOI:** 10.3389/fpls.2020.00450

**Published:** 2020-05-14

**Authors:** Yue Wang, Zhanwu Yang, Youbin Kong, Xihuan Li, Wenlong Li, Hui Du, Caiying Zhang

**Affiliations:** State Key Laboratory of North China Crop Improvement and Regulation, Hebei Agricultural University, Baoding, China

**Keywords:** nodulation, nitrogen fixation, P deficiency, acid phosphatase, APase activity

## Abstract

Nodulation process in legume plants is essential for biological nitrogen fixation during which process a large amount of phosphorus (P) is required. Under P deficiency, nodule formation is greatly affected, and induction of purple acid phosphatases (PAPs) is an adaptive strategy for nodules to acquire more P. However, regulation roles of PAPs in nodules remain largely understood. In this study, by transcriptome sequencing technology, five *PAP* genes were found to be differentially expressed, which led to the greatly increased acid phosphatase (APase) and phytase activities in soybean mature nodules under P starvation conditions; and among the five *PAP* genes, *GmPAP12* had the highest transcript level, and RT-PCR indicated expression of *GmPAP12* was gradually increasing during nodule development. GUS activity driven by *GmPAP12* promoter was also significantly induced in low phosphorus conditions. Further functional analysis showed that under low phosphorus stress, overexpression of *GmPAP12* resulted in higher nodule number, fresh weight, and nitrogenase activity as well as the APase activity than those of control plant nodules, whereas the growth performance and APase activity of nodules on hairy roots were greatly lower when *GmPAP12* was suppressed, indicating that *GmPAP12* may promote P utilization in soybean nodules under low P stress, which thus played an important role in nodulation and biological nitrogen fixation. Moreover, P1BS elements were found in the promoter of *GmPAP12*, and yeast one-hybrid experiment further proved the binding of P1BS by transcription factor GmPHR1 in the promoter of *GmPAP12*. At last, overexpression and suppression of *GmPHR1* in nodules indeed caused highly increased and decreased expression of *GmPAP12*, respectively, indicating that *GmPAP12* is regulated by GmPHR1 in soybean nodules. Taken together, these data suggested that *GmPAP12* was a novel soybean PAP involved in the P utilization and metabolism in soybean root nodules and played an important role in the growth and development of root nodules and biological nitrogen fixation.

## Introduction

Legumes, such as soybean (*Glycine max*), pea (*Pisum sativum*), common bean (*Phaseolus vulgaris*), alfalfa (*Medicago sativa*), and chickpea (*Cicer arietinum* L.), interact with specific soil nitrogen (N)-fixing rhizobia to develop symbiotic relationships that result in the formation of a new organ called root nodules (Oldroyd and Downie, 2008; Ferguson et al., 2010). Soybean, one of the most widely grown legume crops in the world with more than 1,734 million acres of harvest area in 2015, is a very important oil crops that can also provide food, feed, and protein materials (Yuan et al., 2017). Soybean nodules can fix atmospheric N_2_ via rhizobia that provide plants with nitrogen source for growth in rotation systems, which is an efficient way to sustain agricultural system due to symbiotic nitrogen fixation (SNF). Nitrogen is an essential nutrient for plant growth, being one of the important components of amino acid and nucleic acids in the cells. However, the availability of N for plants is very limited in soils that restrict the production of crops. To deal with this situation, agriculture has been largely reliant on nitrogen fertilizers to maximize the crop productivity with about 50% of the nitrogen fertilizers leaching into aquatic system, resulting in environmental pollution. To produce more nitrogen fertilizers, more fossil fuel is used, which costs a heavy price. Thus, it is urgent to find an alternative and effective way to provide nitrogen available for soybean growth; at the moment, biological nitrogen fixation in legume nodules arises as being one of the most hot issues in the world (Oldroyd, 2007; Marx et al., 2016).

SNF of legume nodules is a high-energy-demand process, which requires a large amount of P in energy transfer for optimal nodule functioning than do non-nodulating plants (Makoudi et al., 2018; Ferguson et al., 2019). P is also an essential macronutrient required for optimal plant growth and development. In soil, the low P availability is a critical constriction for plants, especially legume crops in agricultural and natural ecosystems, and is becoming a global problem. Legume nodules are particularly P-rich sinks owing to intensive carbon and energy turnover in which the P content is up to three times that of other plant organs (Schulze et al., 2006; Cabeza et al., 2014). For nodules in particular, the role of P is vital for SNF (Liang et al., 2014; Valentine et al., 2017; Makoudi et al., 2018). Pi deficiency not only affects legume nodule formation and development, nodule number and mass, N_2_ and CO_2_ fixation, and photosynthesis but also N acquisition and metabolism. Great efforts have been made to understand how legumes nodules are responding to P deficiency while symbiotically interacting with rhizobia (Cabeza et al., 2014; Nasr Esfahani et al., 2017). Physiological studies on different legumes plants such as chickpea, common bean, *Medicago truncatula* have shown that legume nodules have developed a series of adaptive strategies that could help conserve P supply to maintain symbiotic activity under Pi starvation conditions (Cabeza et al., 2014; Nasr Esfahani et al., 2017; Isidra-Arellano et al., 2018). These strategies include low P conditions but are not limited to the following: P is preferentially relocated from other organs to nodules to maintain high P content (Tang et al., 2001; Hogh-Jensen et al., 2002); more efficient use of internal phosphate sources to increase P acquisition (Hernandez et al., 2009; Isidra-Arellano et al., 2018), increasing N_2_ fixation of per unit nodule mass while reducing nodule number; and higher oxygen (O_2_) consumption for one unit reduced N_2_ that is related with higher nodule permeability. For nodules, especially in limited P availability conditions, maintenance of P homeostasis is extremely critical for legume growth, development, and symbiosis. Despite that a significant series of studies have done on nodule development and molecular response underlying the adaption of nodules to Pi starvation, it remains a great challenge to explore the molecular and physiological mechanisms that could enable the development of more efficient symbiotic crops for sustainable farming practices (Vance et al., 2003; Schulze, 2004; Sulieman and Tran, 2015).

To cope with P deficiency and stress tolerance for growth, plants have evolved a diverse set of biochemical, physiological, and developmental adaptive strategies that could help their acquisition and utilization of P (Vance et al., 2003). These strategies include alteration of root morphology and architecture, increased activity of high affinity of Pi transporters and acid phosphatases (APases), and accumulation of anthocyanins (Franco-Zorrilla et al., 2004; Jain et al., 2012; Liang et al., 2014). At the molecular level, in order to elucidate the mechanism of plant response to P deficiency, several transcription factors were identified, including MYB, bHLH, GRAS, ERF, and WRKY families (Zhang et al., 2016; Diedhiou and Diouf, 2018; Xue et al., 2018). MYB is one of the largest transcription factor families in the plant kingdom, among which PHR1 (phosphate starvation response regulator (1) functions as a central regular in P starvation signaling, first identified in *Arabidopsis thaliana*. PHR1 binds to the P1BS *cis*-element (5’-GNATATNC-3’), which was prevalently present in the promoters of Pi starvation-induced (PSI) genes (Rubio et al., 2001; Wu et al., 2013; Sun et al., 2016). However, data suggested that expression of *PHR1* was detected in Pi-sufficient conditions and not induced in Pi starvation conditions, indicating that *PHR1* was not or only slightly responsive to Pi starvation (Rubio et al., 2001; Lv et al., 2014; Sun et al., 2016).

Also, it has been reported that under P-deficient conditions, purple acid phosphatases (PAPs) are greatly induced and secreted to improve the acquisition and utilization of Pi. PAPs are a family of metal-containing enzymes found in a wide range of plant species, which exhibit a variety of biological function including P foraging and recycling. In soybean, 23 out of 35 *GmPAP*s were induced by P starvation in different tissues, and nine of them were highly expressed in nodules, indicating that these PAPs may have roles in soybean symbiosis with rhizobia (Li et al., 2012). Recently, soybean *GmPAP21* is found to be induced by P starvation and enhances internal P utilization in nodules, suggesting a role in soybean symbiosis with rhizobia (Yuan and Liu, 2008; Li et al., 2017). Although members of PAPs have been studied in soybean, little is known about the function of PAPs in nodule development and nitrogen fixation under P starvation conditions except *GmPAP21*. Here, we exhibited a low P stress-induced PAP gene, *GmPAP12*, in soybean nodules. Functional analysis by overexpression or suppression of *GmPAP12* in hairy root nodules showed that *GmPAP12* is involved in P homeostasis and nitrogen fixation under P starvation conditions.

## Materials and Methods

### Plant Growth Conditions

Seeds of [*Glycine max* (L.) Merr.] cultivar Williams 82 were surface sterilized and germinated in Petri dishes with wet and sterile filter papers for 3 days under dark conditions in a growth chamber (28°C, 16/8 h light/dark photoperiod). After a 3 days germination, seedlings were transplanted in pots with vermiculite after being inoculated with *Bradyrhizobium diazoefficiens* USDA 110 (*Bradyrhizobium japonicum* USDA 110). Until the first trifoliate leaves were fully developed, soybean plants were watered with nitrogen-free nutrient solution containing 5 μM (low P:LP) and 500 μM (high P:HP) of KH_2_PO_4_, respectively. At 10, 17 days after inoculation with *B. diazoefficiens* USDA 110, only nodules were harvested. At 28 days, soybean leaves, roots, and nodules were separately harvested for measuring fresh weight, dry weight, height of shoot and root, total P and N content, nodule number and nodule size, phytase and phosphatase activities, and acetylene reduction assay; nodule size was calculated as the average fresh weight of a single nodule. For dry weight and total P and N content, samples were oven-dried, and other fresh samples were stored at −80°C for RNA extraction and quantitative real-time PCR (qRT-PCR) analysis.

### RNA Isolation and RNA-Seq

Twenty-eight-day nodules collected with three independent biological replicates for each Pi treatment were ground in liquid nitrogen, and total RNA was extracted using TRIzol reagent (Invitrogen, United States). RNA purity was checked using the NanoPhotometer^®^ spectrophotometer (IMPLEN, CA, United States), and RNA concentration was measured using Qubit^®^ RNA Assay Kit in Qubit^®^ 2.0 Flurometer (Life Technologies, CA, United States). RNA samples were treated with RNase-free DNase I (TaKaRa, Tokyo, Japan) to avoid genomic DNA contamination. cDNA was transcribed using a Prime Script^TM^ RT reagent Kit (Perfect Real Time) with gDNA Eraser (TaKaRa Bio, Inc.). Six cDNA libraries were generated and sequenced on an Illumina NovaSeq 6000 platform. After quality control, a total of 25,155,773 clean reads were analyzed for differential expression of two conditions, which was performed using the DESeq R package (1.18.0). FPKM method was used to estimate gene expression levels. The resulting *p*-values were adjusted using the Benjamini and Hochberg approach for controlling the false discovery rate. Genes with an adjusted *p* < 0.05 found by DESeq were assigned as differentially expressed genes (DEGs).

### Quantitative Real-Time PCR

qRT-PCR was performed using SYBR Premix EX Tag^TM^ (TaKaRa) on a CFX96^TM^ real-time system (Bio-Rad). The soybean *Actin11* gene was used as an endogenous control to normalize the other samples. The specific primers used are shown in Supplementary Table S2. The qRT-PCR conditions were as follows: 30 s at 95°C followed by 40 cycles of 5 s at 95°C, 15 s at 60°C and 12 s at 72°C, and a final 5 s at 72°C. The cycle threshold (CT) values of each sample were standardized using *Actin11* gene, and the relative changes of gene expression were analyzed using the 2^–ΔΔ*CT*^ method (Livak and Schmittgen, 2001).

### Construction of *GmPAP12* Promoter, Overexpression, and RNAi Constructs and Soybean Hairy Root Transformation

For the analysis of the *GmPAP12* promoter, 2,000-bp fragment upstream of *GmPAP12* transcription start codon was generated by PCR and cloned into the pBI121 vector between *Hin*dIII and *Bam*HI restriction enzyme site in-frame with *GUS* reporter gene to make *pGmPAPA12-GUS* construct. To generate overexpression construct, full-length open reading frame (ORF) of *GmPAP12* or *GmPHR1* was amplified using PCR from soybean root nodule cDNA and then cloned into pCAMBIA1390 under *CaMV 35S* promoter. For *RNAi* constructs, about 220 bp of sense and antisense fragments targeting *GmPAP12* or *GmPHR1* was cloned into pTCK-303 vector under *CaMV 35S* promoter (Du et al., 2016). Also, the corresponding constructs were transformed into *Agrobacterium rhizogenes* strain K599 for hairy root transformation. Transgenic hairy roots emerging from K599 infection site were examined for GUS expression. Only one GUS positive root was left, and all the other roots were cut in the plant root system. Then the plants were inoculated with *B. diazoefficiens* USDA 110 and grown in two different P conditions. At 28 days after rhizobium inoculation, nodules were harvested and analyzed for nodule number, nodule weight, nitrogenase activity, RNA isolation, N and P contents, and APase activity measurement (Kim et al., 2013; Li et al., 2017).

### Acetylene Reduction Assay

Nitrogenase activity was measured by acetylene reduction assay; the method was used as described previously (Oh et al., 2001).

### Measurement of N and P Contents

First, samples were dried, weighed, ground into fine powder, and then digested with HNO_3_ in a microwave oven; the resulting samples were used for determination of N and P contents. P content was measured by the color reaction of P-molybdate blue at absorbance of 700 nm (Grunwald et al., 2009). N content was measured using semimicro-Kjeldahl determination method in a nitrogen analyzer.

### Acid Phosphatase and Phytase Activity Measurements

APase activity of nodules was determined by measuring the amount of nitrophenol from *p*-nitrophenyl phosphate (*p*-NPP). Total nodule protein extracts were mixed with Na-acetate buffer containing 1 mM of *p*-NPP and incubated at 37°C for 30 min; the reaction was stopped by adding 1 M of NaOH. The absorbance was measured at 405 nm. APase activity was expressed as micromoles of *p*-NPP per minute per milligram of protein.

For phytase activities, total nodule proteins were added to a mixture containing 1 mmol/L of phytate and then incubated with malachite green reagent; the optical density (OD) values were measured at 650 nm. Phytase activity was calculated as micromoles of Pi released per minute per milligram of protein (Kong et al., 2019). All experiments were triplicate with five independent samples per replicate.

### Yeast One-Hybrid Assay

Yeast one-hybrid (Y1H) experiment was performed using Matchmaker Gold Systems (Clontech). *GmPHR1* was cloned into PGADT7-AD vector (TaKaRa), and the resulting construct was named PGADT7-AD-*GmPHR1*. Three P1BS sequences were found in the promoter of *GmPAP12*: 5’-GTATATTC-3’, 5’-GCATATTC-3’, and 5’-GAATATTC-3’. Three tandem repeats of each P1BS element were synthesized and cloned into pAbAi vector (TaKaRa) used as baits. The consequent pAbAi-3xP1BS vectors were digested with *Bst*BI (NEB, New England Biolabs) restriction enzyme and then transformed into Y1H cells containing PGADT7-AD (negative control) or PGADT7-AD-GmPHR1, respectively. The transformants were grown on selective medium SD-Leu containing antibiotic aureobasidin A (AbA) (250 μg/ml) for 4 days at 30°C. AbA is used as a stringent, highly selective reporter.

### Histochemical GUS Staining Assay

To detect GUS activity in transgenic soybean root nodules, fresh root tissues were incubated at 37°C for 12 h in 5-bromo-4-chloro-3-indolyl-β-D-glucuronic acid (X-Gluc) containing solution (Zhong et al., 2013). After X-Gluc incubation, the root tissues were washed with ethanol (70% v/v), and then photographs were taken.

### GUS Activity Assay

Total proteins of transgenic soybean root nodules were extracted and quantified as previously described (Freitas et al., 2019). GUS assay was performed in a mixture containing 10 mM of 4-methylumbelliferyl-β-D-glucuronide (MUG; Sigma, United States), and the mixture was incubated for 1 h at 37°C. The fluorescence product of 4-methylumbelliferone (4-MU) was monitored using a VersaFluor Fluorometer (Bio-Rad) with excitation at 365 nm and emission at 455 nm. GUS activity was calculated in picomoles of MU produced per minute per microgram of soluble protein. The assay was repeated at least three times. The results were shown as the mean of independent experiments with the respective standard deviation. Asterisks (^∗∗^) above the bars indicate significant differences at *p* < 0.01.

### Statistical Methods

Statistical analyses were performed using SPSS 17.0 software (IBM, United States).

## Results

### Soybean Performance Was Significantly Reduced Under P-Deficient Conditions

The performance of soybean plants was assessed at 28 days post inoculation with rhizobium *Bradyrhizobium diazoefficiens* USDA 110 when the nodules of soybean are mature and are responsible for nitrogen fixation (Cheng et al., 2011; Ferguson et al., 2010; Zhang et al., 2014). In low P treatment, soybean growth was significantly affected with reduced plant height and yellow leaves in the base of the soybean plant (Supplementary Figure S1A). The root-to-shoot ratio of soybean plants was increased in the P-deficient supply, which was consistent with previously data in other plant species in low P conditions (Supplementary Figure S1B) (Lopez-Bucio et al., 2003; Gruber et al., 2013; Sun et al., 2014). Consequently, plant shoot and root growth were decreased in low P stress leading to reduced total (shoot, and root plus nodule) fresh weight and dry weight production by 29.2 and 26.2%, respectively, when compared with those in high P conditions (Supplementary Figures S1C,D). All these data suggested that P deficiency greatly affects soybean plant growth and biomass production in decreased P supply.

**FIGURE 1:**
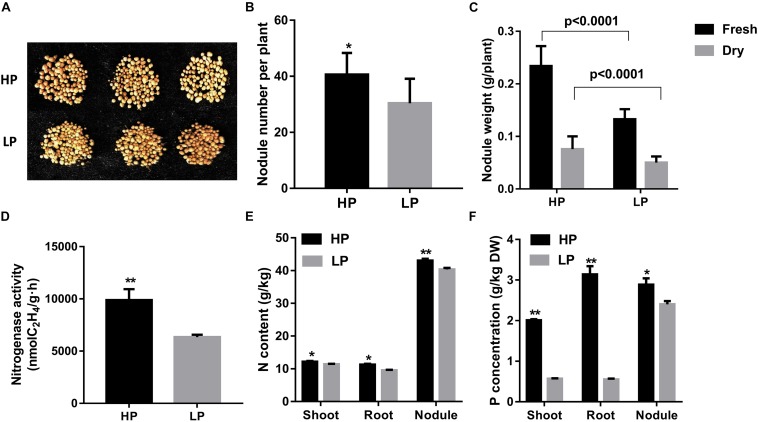
Nodulation parameter analysis of soybean plant nodules treated by different P levels. **(A)** Photographs of plant nodule growth performance. **(B)** Nodule number. **(C)** Nodule total weight per plant. **(D)** Nitrogenase activities of nodules measured by acetylene reduction assay. **(E)** Nodule N content. **(F)** Nodule P content. Soybean plant samples were harvested 28 days after inoculation with rhizobium watered with nitrogen-free nutrient solution. Data are presented as the average of three different biological replicates and 20 plants for each replicate. Bars show the means ± SD values. Asterisks indicate significant difference within a P level in *t*-tests. *p < 0.05, **p < 0.01.

Next, nodulation parameters were investigated here to know the effect of low P supply on nodule organogenesis. Significantly reduced soybean nodulation was observed in soybean plants under low P conditions (Figure 1A). Nodule number and nodule fresh and dry weight accumulation were greatly decreased by 25.0, 41.6, and 33.3%, respectively, in P-deficient conditions compared with high P conditions (Figures 1B,C). Moreover, nitrogenase activity of nodules was dramatically affected and reduced by 58% in low P stress conditions (Figure 1D). These results further illustrated that low P supply affected nodule growth and development as well as N_2_ fixation in soybean. Next, N assimilation was evaluated in different organs (shoot, root, and nodules) and was found to be decreased under P depletion stress (Figure 1E). P content in shoot and root was dramatically reduced by 72.5 and 82.2% respectively, although in nodules, it was decreased by only 20.8%, which was less strong in low P stress than in high P conditions (Figure 1F). These data suggested that low P supply affected soybean nodulation and that P homeostasis was very important for soybean growth and symbiotic N_2_ fixation.

### Purple Acid Phosphatase Genes Were Highly Induced in Low P Stress in Soybean Nodules

**FIGURE 2:**
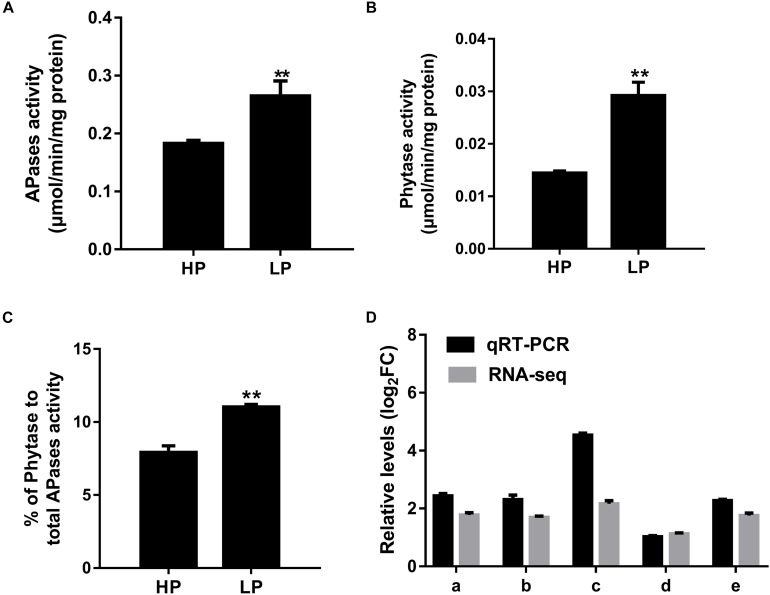
Relative transcript levels and phytase and APase activities of *GmPAP*s in nodules obtained in low P (LP) and high P (HP) conditions. **(A)** Nodule APase activity. **(B)** Nodule phytase activity. **(C)** Percentage of phytase activity to total APases of nodules grown under different P conditions. Asterisks indicate significant difference within a P level in *t*-tests. **p < 0.01. **(D)** A heatmap presentation of fold changes of *PAPs* identified from RNA-seq and qRT-PCR in LP conditions compared with HP conditions. qRT-PCR was performed using three different biological samples with three repeats for each sample. Phytase and APase activities were analyzed from four independent experiments, and average results were shown. For each repeat, five plants were used. (a) *Glyma.06G170300*-LP/*Glyma.06G170300-*HP, expression of *Glyma.06G170300* in nodules under P-deficient condition vs. P-sufficient conditions; (b) *Glyma.08G093500*-LP/*08G093500-HP*, expression of *Glyma.08G093500* in nodules under P-deficient condition vs. P-sufficient conditions; (c) *Glyma.06G028200*-LP/*06G028200-*HP, expression of *Glyma.06G028200* in nodules under P-deficient conditions vs. P-sufficient conditions; (d) *Glyma.05G247800*-LP/*Glyma.05G247800-*HP, expression of *Glyma.05G247800* in nodules under P-deficient condition vs. P-sufficient conditions; (e) *Glyma.07G191500*-LP/*Glyma.07G191500-*HP expression of *Glyma.07G191500* in nodules under P-deficient condition vs. P-sufficient conditions.

Under low P stress, APases and phytase are always induced and secreted. Here, with regard to APase and phytase activities of soybean nodules under low P conditions, we found that APase and phytase activities were greatly increased by about 50.0 and 100%, respectively, than in high P conditions (Figures 2A,B). Further, the percentage of phytase activity to total APase activity was also raised by 36.6%, which indicates increased PAP activities under phosphorus deficiency (Figure 2C).

In order to investigate the mechanisms at molecular level for the enhanced APase and phytase activities in soybean nodules under low P conditions, RNA sequencing was conducted to elucidate the transcriptome of soybean mature nodules. As expected, five PAP genes were greatly up-regulated, which could explain the increased phytase and APase activities indicating importance of Pi remobilization and recycling activities in soybean nodules under low P conditions (Table 1). Transcripts of these PAPs in nodules were verified via qRT-PCR, and all the PAPs here were largely induced under P starvation conditions compared with high P conditions, which was consistent with RNA-seq results (Figure 2D).

**TABLE 1:** Differentially expressed *GmPAP*s induced by low P stress in nodules.

	Gene_id	log_2_ fold	*p*-value	*p*-adj
		change		
GmPAPs	Glyma.06G170300	1.7044	3.59E–20	2.67E–16
	Glyma.08G093500	1.6811	2.67E–09	1.15E–06
	Glyma.05G247800	1.1965	6.40E–09	2.35E–06
	Glyma.07G191500	1.1877	1.90E–05	0.0016919
	Glyma.06G028200	1.9881	0.00010032	0.016771

*E = 10, E–20 = 10^–20^; pval = p-value; padj = p adjust = q-value.*

### *GmPAP12* Plays Essential Roles in Nodule Development Under P Deficiency

**FIGURE 3:**
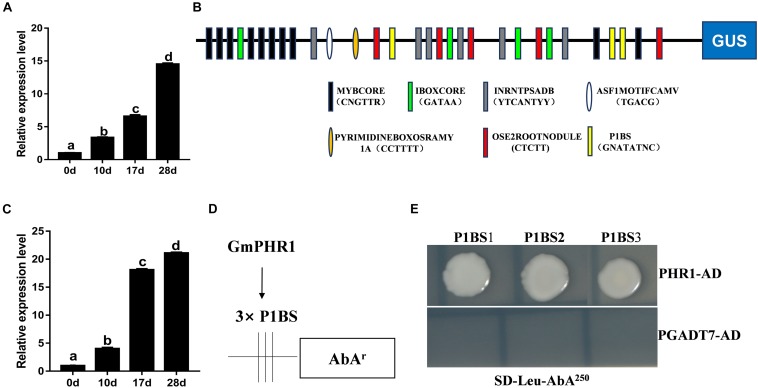
Promoter analysis of *GmPAP12* in transgenic soybean nodules. **(A)** Expression of *GmPAP12* in nodules determined by qRT-PCR. **(B)** *cis*-Elements of *GmPAP12* promoter analyzed by PlantCARE. **(C)** Expression of *GmPHR1* in nodules determined by qRT-PCR. Nodules were harvested at 0, 10, 17, and 28 days post rhizobium inoculation in normal growth condition. **(D)** The diagram shows the construct of *GmPAP12* promoter for yeast one-hybrid (Y1H). **(E)** Y1H results. Three tandem repeats of each P1BS element were used as baits and cloned into pAbAi vector. The resulting pAbAi-3xP1BS vectors were transformed into Y1H cells expressing PGADT7-AD (negative control) or PGADT7-AD-GmPHR1, respectively. The transformants were grown on selective medium SD-Leu containing antibiotic AbA (250 μg/ml), and pictures were taken after 4 days of incubation at 30°C. The Y1H assays were repeated three times. Letters in **(A,C)** indicate significant differences (one-way ANOVA, *p* < 0.05).

In order to explore the effects of PAPs on soybean nodulation further, *Glyma.06G028200*, which was named *GmPAP12* according to the genome of soybean (the Phytozome website)^1^, was chosen for further study owing to its highest expression level among these five PAPs in nodules induced by low P stress. First, the expression pattern of *GmPAP12* was checked and increasingly expressed during nodule development in normal growth conditions, which suggested that *GmPAP12* might be involved in soybean nodulation and nitrogen fixation (Figure 3A). Next, promoter of *GmPAP12* was analyzed using a promoter analysis program PlantCARE, and several *cis*-elements within 2-kb sequence upstream of start codon were found to respond to stress, gibberellin, photosynthesis, light, and so on (Supplementary Table S1 and Figure 3B). Three P1BS *cis*-elements present in the promoter of *GmPAP12* suggested that GmPHR1 may directly bind to and regulate *GmPAP12*. To test this hypothesis, *Glyma.19G122700* (Accession No. HQ007311) named *GmPHR1* in our previous study was used for further research (Li et al., 2014). qRT-PCR showed that the transcript pattern of *GmPHR1* was similar with that of *GmPAP12* during the process of nodule growth (Figure 3C). A Y1H assay was performed against all the three P1BS sequences from *GmPAP12* promoter, and results showed that GmPHR1 can directly bind to all the three P1BS elements, indicating the regulation of *GmPAP12* by GmPHR1 in the transcript level during nodule development (Figures 3D,E).

**FIGURE 4:**
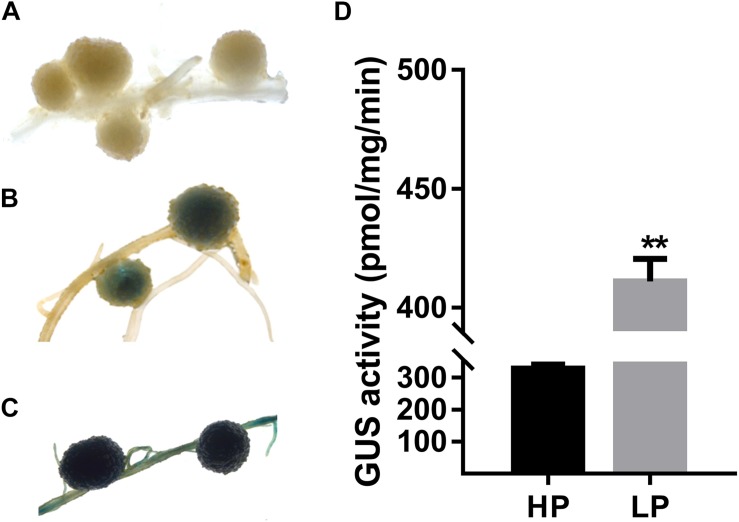
Promoter analysis of *GmPAP12* gene in transgenic soybean nodules. **(A)** Histochemical GUS staining of wild-type (WT) nodules growing in low P (LP) conditions. **(B)** Histochemical GUS staining of *GmPAP12* promoter transgenic nodules growing in high P (HP) conditions. **(C)** Histochemical GUS staining of *GmPAP12* promoter in transgenic nodules growing in LP conditions. Promoter of *GmPAP12-GUS* transgenic composite soybean plants was treated with different P levels, and nodules were harvested 28 days after inoculation with rhizobia for GUS staining. **(D)** GUS activity of *GmPAP12* promoter in transgenic nodules. Values are means of 10 independent lines for each P treatment. Bars show the means ± SD values. Asterisks indicate significant difference within a P level in *t*-tests. **p < 0.01.

**FIGURE 5:**
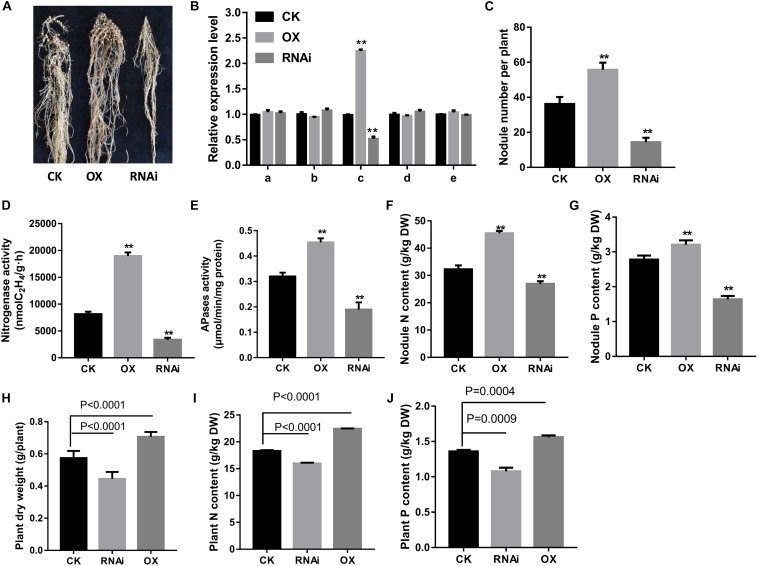
Effects of *GmPAP12* on nodulation in *GmPAP12* overexpressing (OX) or suppressing (*RNAi*) transgenic composite lines. **(A)** Nodule performance. **(B)** Relative transcript levels of PAPs in nodules. **(C)** Nodule number. **(D)** Nitrogenase activity. **(E)** APase activity. **(F)** Nodule N content. **(G)** Nodule P content. **(H)** Plant shoot dry weight. **(I)** Plant N content. **(J)** Plant P content. *GmPAP12* OX or RNAi transgenic composite plants were examined 28 days after inoculation with rhizobia in low P conditions watered with N-free nutrient solution. Data are presented as the average of three different biological replicates and 20 plants for each replicate. Bars show the means ± SD values. Asterisks indicate significant difference within a P level in *t*-tests. **p < 0.01. CK means control.

Next, expression of *GmPAP12* was confirmed by *GmPAP12* promoter:*GUS* construct in transgenic composite root nodules under P starvation conditions. Visual GUS staining was observed in transgenic mature nodules with about 36.5% higher GUS activity in low P stress than in P-sufficient conditions (Figure 4). Furthermore, the functional analysis of transgenic composite root nodules either overexpressing (OX) or suppressing (RNAi) *GmPAP12* was generated, and the related phenotypes were evaluated in low P conditions only (Figure 5A). First, qRT-PCR demonstrated that the transcript level of *GmPAP12* in OX transgenic root nodules was about 120% higher, whereas in *GmPAP12*, RNAi transgenic nodules showed only 50% lower expression than those of control (CK) lines under P-deficient conditions, while the expression of the other four PAP genes was unchanged (Figure 5B). The total nodule number, and nodule nitrogenase and APase activities of *GmPAP12* OX composite lines were increased by 44.8, 100, and 38.7%, respectively, resulting in increased N content by 17.0% and P content by 19.0%, whereas in *GmPAP12*, RNAi composite lines, the total nodule number, and nitrogenase and APase activities decreased by 51.2, 50.0, and 40.1%, respectively, when compared with those of control lines in low P stress, resulting in decreased N and P contents by 33.6 and 37.0%, respectively (Figures 5C–G). These data indicated that *GmPAP12* can enhance P utilization efficiency and nodule nitrogen fixation. Next, the effect of low P stress on *GmPAP12* OX or RNAi whole transgenic composite plants was analyzed. The plant shoot dry weight and N and P contents in *GmPAP12* OX transgenic composite plants were increased by 22.9, 22.8, and 14.8%, respectively, whereas *GmPAP12* RNAi transgenic composite plant shoot dry weight and N and P contents were decreased by 22.5, 12.6, and 14.8% compared with those of control plants in low P stress. All these data indicated that altering *GmPAP12* gene expression could significantly influence soybean nodulation and nitrogen fixation capacity as well as plant growth performance in low P stress (Figures 5H–J).

### *GmPAP12* Is Mainly Regulated by GmPHR1 Under P Starvation Conditions

**FIGURE 6:**
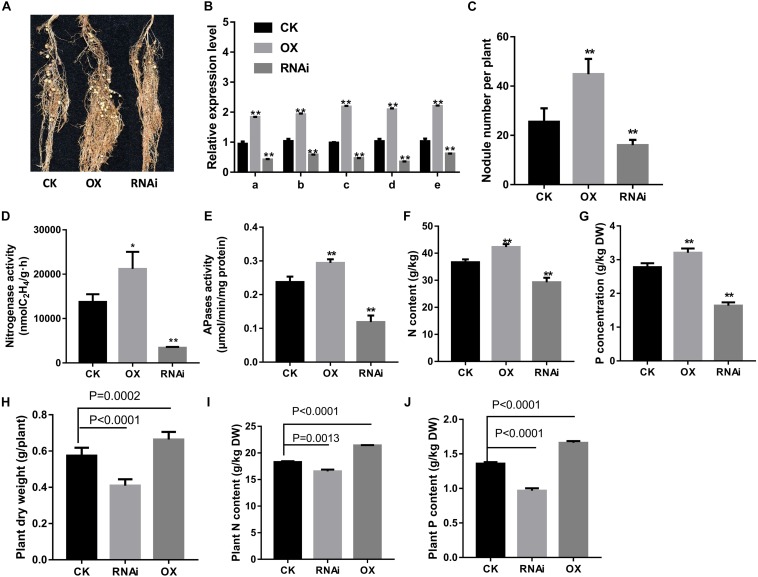
Effects of *GmPHR1* on nodulation in *GmPHR1* overexpressing (OX) or suppressing (*RNAi*) transgenic composite lines. **(A)** Nodule performance. **(B)** Relative transcript levels of PAPs in nodules. **(C)** Nodule number. **(D)** Nitrogenase activity. **(E)** APase activity. **(F)** Nodule N content. **(G)** Nodule P content. **(H)** Plant shoot dry weight. **(I)** Plant N content. **(J)** Plant P content. *GmPAP12* OX or RNAi transgenic composite plants were examined 28 days after inoculation with rhizobia in low P conditions watered with N-free nutrient solution. Data are presented as the average of three different biological replicates and 20 plants for each replicate. Bars show the means ± SD values. Asterisks indicate significant difference in LP conditions in *t*-tests. *p < 0.05, **p < 0.01. CK means control.

Next, in order to elucidate the regulation of *GmPAP12* by GmPHR1 underlying transcript response to low P stress in nodules, a function of GmPHR1 was further analyzed in transgenic composite soybean plants. It showed significantly increased and decreased expression of *GmPAP12* as well as the other four PAPs in *GmPHR1* OX and RNAi transgenic composite nodules, respectively, compared with control nodules in low P stress. The *GmPHR1* OX lines showed significantly improved nitrogen fixation capacity proved by increased nodule performance, nodule number, and nitrogenase and APase activities, which led to the significantly increased N and P contents, whereas *GmPHR1* RNAi lines had much lower nitrogen fixation than had control nodules in low P conditions (Figures 6A–G). These data indicated that *GmPHR1* can also enhance P utilization efficiency and nodule development and regulate the expression of these five PAPs. Next, the effect of low P stress on *GmPHR1* OX or RNAi whole transgenic composite plants was analyzed. The plant shoot dry weight and N and P contents in *GmPHR1* OX transgenic composite plants were increased by 15.7, 17.1, and 22.2%, respectively, whereas *GmPHR1* RNAi transgenic composite plants showed decreased plant shoot dry weight and N and P contents by 28.7, 9.6, and 24.6% than did control plants in low P stress. All these data indicated that altering *GmPHR1* gene expression could significantly influence soybean nodulation and nitrogen fixation capacity as well as plant growth performance by regulating PAPs in low P stress. Also these data further illustrated that *GmPAP12* facilitates nodule development as well as nitrogen fixation mainly regulated by GmPHR1 under low P conditions.

## Discussion

Nitrogen is a component of biological molecules in plants, which are critical for sustained plant development. In agriculture, nitrogen available is becoming a constraint on crop yield and reproduction. Although there is plenty of nitrogen in the atmosphere, it is not directly available for plants. Legume plants such as soybean can have a symbiotic association with nitrogen-fixing bacteria called rhizobia, resulting in the formation of nodule, which is responsible for biological nitrogen fixation. P deficiency significantly affected N_2_ fixation documented in nodules of chickpea, *Medicago truncatula*, soybean, and common bean. Additionally, the secretion of PAPs in plants is a widely known as an adaptation strategy in response to P deficiency. Under low P conditions, in nodules of *M. truncatula*, chickpea, and soybean, they were found to be differentially upregulated by RNA-seq transcriptome analysis, which indicates that nodules acclimated to P deficiency by increasing P turnover (Cabeza et al., 2014; Nasr Esfahani et al., 2017; Xue et al., 2018).

In this work, consistent with the significantly increased APase activity in soybean nodules under P deficiency, five PAP genes were found among the DEG in RNA-seq data, indicating that PAPs play vital role in P metabolism and mobilization in soybean nodules (Table 1 and Figure 2D). Although functions of PAPs in soybean have been studied, including *GmPAP3* and *GmPAP4*, roles of P starvation-induced PAPs in nodules need to be more explored. Recently, *GmPAP21* was proved to be highly induced in nodules by P limitation, which indicated that the involvement of *GmPAP21* in internal P metabolism and overexpression of *GmPAP21* significantly decreased plant dry weight and N and P contents, thus inhibiting nodule growth (Li et al., 2017). Here, expression of *GmPAP12* was notably increased during nodule development, and GUS activity driven by the promoter of *GmPAP12* was also more induced in nodules as well as roots under P starvation compared with P-sufficient conditions (Figures 3A, 4). Furthermore, the functional analysis of transgenic hairy root nodules in *GmPAP12* OX lines showed increased plant shoot dry weight and N and P contents, which indicated that *GmPAP12* promoted nodule development and nitrogen fixation under low P conditions (Figure 5). These data indicate that *GmPAP12* is involved in P utilization and integrates the SNF and P metabolism signaling in nodules under P starvation situation.

Nodules require much more P for legume nodulation and SNF, so the P content in nodules is relatively high. Under low P conditions, P is preferentially relocated from other organs to nodules to maintain nitrogen fixation, indicating that P homeostasis is very important in the nodule. In soybean, in low P stress growing in hydroponic conditions, P content in both leaves and roots are 10 times higher in high P than in low P treatment, whereas in nodules, only 1.75 times are increased (Sulieman et al., 2010; Qin et al., 2012). In this study, with regard to P content, it was much less affected in nodules than in shoot and root in P deficiency, suggesting that nodules represent a P sink to maintain Pi homeostasis and the ability of N_2_ fixation during nodule development; it is hypothesized that nodules try to regulate N_2_ fixation process to adaptation to low P stress by allocating P from other organs to nodules (Cabeza et al., 2014; Xue et al., 2018).

Previous research also showed that increase in the exudation and APase activity of PAPs is one of the strategies for plants to increase P recycling in nodules (Li et al., 2012). Also, in nodules of common bean at limited P conditions, activities of APase increased, indicating that nitrogen fixation can enhance P utilization in nodules in response to P deficiency (Araújo et al., 2008; Mandri et al., 2012; Makoudi et al., 2018). In this study, APase activities of nodules in low P conditions were also greatly increased in *GmPAP12* OX transgenic composite nodules, indicating that *GmPAP12* is responsible for P homoeostasis in nodules (Figure 5A). In high P conditions, *GmPAP12* showed increasing expression during nodule development, which suggested that *GmPAP12* might be involved in nodulation and nitrogen fixation beyond P recycling (Figure 3A). All these data suggest that GmPAP12 plays a role in nodulation and sustains nitrogen fixation in low P conditions.

PHR1, a transcription factor mainly studied well in *Arabidopsis* and rice, is playing a key role in P starvation signaling by binding to the P1BS element in the promoters of PSI genes (Rubio et al., 2001; Zhou et al., 2008). Under P-deficient conditions, overexpression of PHR1 results in induction of PSI genes and cellular P accumulation, whereas loss of function of PHR1 reduces expression of PSI genes and accumulation of anthocyanins, which are typical symptoms in plants under P starvation (Nilsson et al., 2007). Recently, in *Arabidopsis*, evidence indicates that P starvation-induced APase *AtPAP10* was regulated by AtPHR1, which binds to the only P1BS element of *AtPAP10* promoter. In this study, the promoter analysis indicates that *GmPAP12* contains three P1BS elements, and Y1H confirmed that GmPHR1 can bind to all the three P1BS elements (Figures 3D,E and Supplementary Table S1). In *GmPHR1* OX or RNAi transgenic nodules, expression of *GmPAP12* was also greatly affected under P starvation conditions. These data indicate under P starvation conditions that *GmPAP12* functions in nodulation and that nitrogen fixation is transcriptionally regulated by *GmPHR1* (Figure 3E). Also, expression of *GmPHR1* was gradually increasing in nodules in normal growth conditions, indicating that *GmPHR1* functions in nodule development also beyond P utilization. In *GmPHR1* OX or RNAi transgenic composite plants, plant shoot dry weight and N and P contents were increased or decreased, respectively, which suggested that *GmPHR1* is involved in nitrogen fixation process dealing with low P stress (Figures 3C, 6).

Previous evidence indicates that PHR1 was expressed in normal conditions and not responsive to P starvation (Rubio et al., 2001; Zhou et al., 2008). Also, their function in P signaling is modulated by SPX4 protein regulating the targeting of PHR1 from cytoplasm to nucleus (Lv et al., 2014). In our mRNA-seq data, several *Arabidopsis PHR1* homolog *PHR1* genes were identified in soybean nodules, and none of them showed response to Pi starvation. Expression of five *GmPHR1* genes was confirmed by RT-PCR, and also no significant expression changes were detected between P-sufficient and P-deficient conditions (Supplementary Figure S2). This suggested in nodules that it also has SPX-containing proteins or others to interact with PHR1 to regulate downstream PSI genes such as *GmPAP12*, so our further study is going to identify interacting partners of PHR1 in nodules and explore more about the molecular mechanism underlying the relationship between nitrogen fixation capacity and P availability, which will help the development of more efficient soybean plants.

## Data Availability Statement

The datasets generated for this study can be found in the NCBI BioProject, https://www.ncbi.nlm.nih.gov/bioproject/PRJNA605671.

## Author Contributions

CZ, HD, YW, and ZY conceived of and designed the research. YW, ZY, and HD performed the entirety of the experiment and analyzed the data. HD wrote the manuscript. XL and WL provided suggestions during all the processes of the experiments. All authors participated in the revision of the manuscript.

## Conflict of Interest

The authors declare that the research was conducted in the absence of any commercial or financial relationships that could be construed as a potential conflict of interest.

## Footnotes

1 https://phytozome.jgi.doe.gov/pz/portal.html
