# Supplementary material for: GmPAP12 Is Required for Nodule Development and Nitrogen Fixation Under Phosphorus Starvation in Soybean
Source: Front Plant Sci. 2020 May 14;11:450. doi: 10.3389/fpls.2020.00450 (PMC7243344; doi:10.3389/fpls.2020.00450)
Supplement: Supplementary file 1 [file Data_Sheet_1.docx]

**Supplementary Material**

**GmPAP12 is required for nodule development and nitrogen fixation under phosphorus starvation in soybean**

**Tables**

**Table S1.** **Cis-acting elements located in the promoters of 2-kb upstream of *GmPAP12* start codon.**

| **Cis-acting elements** | **Sequences** | **Functions** |
| --- | --- | --- |
| P1BS | GNATATNC | PHR1 or PHR1-like binding site |
| OSE2ROOTNODULE | CTCTT | Nodule development |
| MYBCORE | CNGTTR | Dehydration stress |
| PYRIMIDINEBOXOSRAMY1A | CCTTTT | Gibberellin-Responsive |
| INRNTPSADB | YTCANTYY | Photosynthesis |
| IBOXCORE | GATAA | Light-regulated transcription |
| ASF1MOTIFCAMV | TGACG | Disease resistance |

**Table S2. Primers used for RT-PCR.**

| Gene ID | Forward primer | Reverse primer |
| --- | --- | --- |
| Glyma.03G143600 | ACCAAGTTCCTTCCGGTTGA | TACGGCCGCCACATTAGAAG |
| Glyma.09G113000 | AGTTAGATGTTCAGCGGCGT | CCCGTCAGAAGTGGAAACCA |
| Glyma.9G113100 | TTGTGGCGGTCAATAGCCTT | TTCCGTGACCATGTCTCTGC |
| Glyma.13G126200 | AGTGAACCATGGCAGGTCAG | CAGTTCTTTGACGCTTCGGG |
| Glyma.12G104600 | CAGATGCTACCCAGATCCATGA | TTCTGCCACGGAGTGAACAA |
| Glyma.06G092000 | CTATCCACAGTAAACACCG | ACCATTGATTAAACCTCGCT |
| Glyma.02G124300 | TAGATCTCCATGCTATCCTA | TGGCGACTACAAGCATAAC |
| Glyma.06G103300 | GTCATGTTGCAACCAGCT | CAAGATCAAGCGTGTGATC |
| Glyma.10G146300 | CCCGGCGATCTCGATTTC | GCCTTCGCCTTCTTGTGCT |
| Glyma.11G194100 | CCATGCAATGCTATTACCCAAT | AGCTGTGGCAGCCAATTCTT |
| Glyma.17G155900 | AGACACAGACCTTGCAACAG | GGTGTTGGTGTTGTCAAAA |
| Glyma.20G231800 | CAATCACAAGACCCTTTTGG | TCTAAGGTGTTACTGGGGTC |
| Glyma.06G170300 | CAAGACCGTTTTGGCGCTAC | ATGGTTCCTCTACCCAGCCA |
| Glyma.08G093500 | AAGGACGTGGATTTGGCACT | AAGCAGTGATCGTGTCCGTT |
| Glyma.06G028200 | CCAACAGGACGCTTACCCAT | TTCCCGCAGTCCAAATCCAA |
| Glyma.05G247800 | TGGGTTTCGTTAGCCTGTGT | AACTTTGCAGCAACCCTTCCC |
| Glyma.07G191500 | CATGCAGCCGGTGCTATCAT | GCCCAACTAGCATCAACAGC |
| Actin11 | ATCTTGACTGAGCGTGGTTATTCC | GCTGGTCCTGGCTGTCTCC |

**Fig. S1**

**
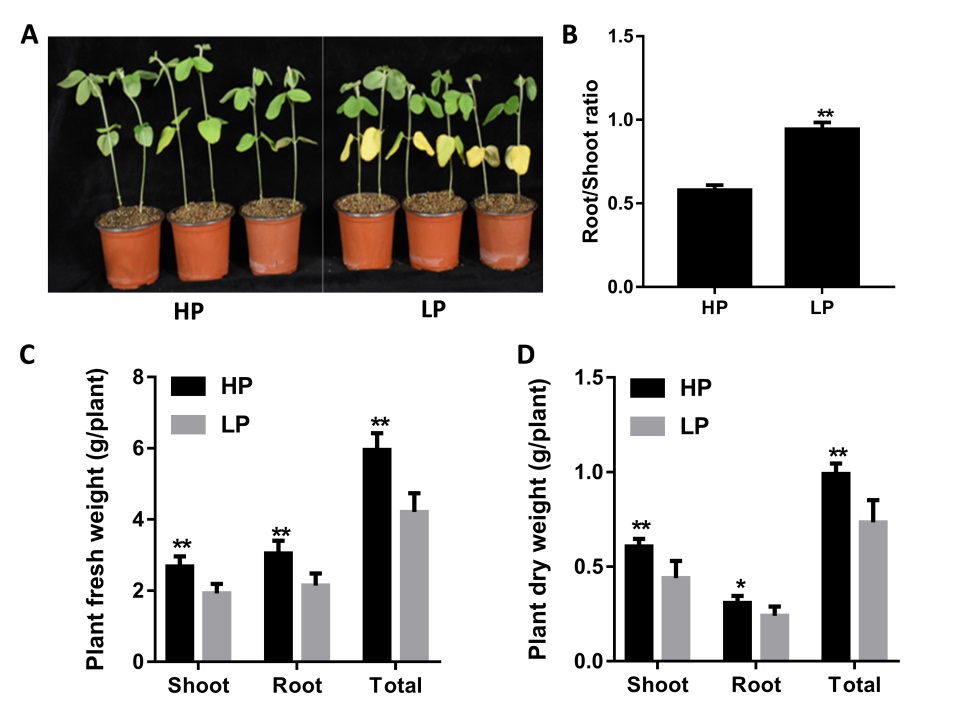
**

**Figure. S1** **Effects of low P stress on soybean growth phenotype. (A)** Photographs of plant performance. **(B)** Root to shoot ratio. **(C)** Plant fresh weight. **(D)** Plant dry weight. Seedlings were grown at P deficient (LP, 500μM KH_2_PO_4_) and sufficient (HP, 5mΜ KH_2_PO_4_) conditions and harvested 28 days after inoculated with rhizobia. Data are means of four replicates, and error bars show the SE values. Asterisks in B-D indicate significant difference between different P level in *t* test. * p<0.05, ** p<0.01.

**Fig. S2**

**
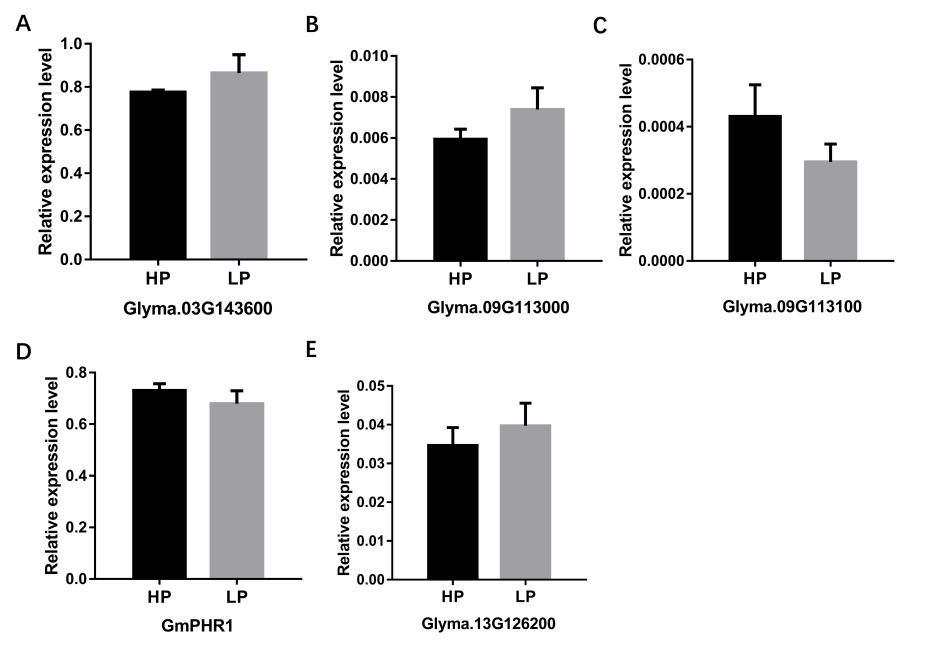
**

**Figure. S2 Expression of five PHR1 genes detected by qRT-PCR.** qRT-PCR was performed using three different biological samples with three repeat for each sample and the data are presented here as the means± SD. A: *Glyma.03G143600*, B: *Glyma.09G113000*, C: *Glyma.09G113100*, D: *GmPHR1*, E: *Glyma.13G126200.*
